# Intensive care unit course of infants and children after cranial vault reconstruction for craniosynostosis

**DOI:** 10.1186/1756-0500-4-347

**Published:** 2011-09-09

**Authors:** Olugbenga A Akingbola, Dinesh Singh, Sudesh K Srivastav, John W Walsh, David A Jansen, Edwin M Frieberg

**Affiliations:** 1 Department of Pediatrics 1430 Tulane Avenue, New Orleans, LA 70112, USA; 2 Tulane Institute of Public Health and Tropical Medicine 1440 Canal Street, New Orleans, LA 70112, USA; 3 Departments of Neurosurgery and Plastic Surgery, Tulane University, New Orleans, LA 70112, USA

## Abstract

**Background:**

Craniosynostosis (CSS) results from the premature closure of one or more cranial sutures, leading to deformed calvaria at birth. It is a common finding in children with an incidence of one in 2000 births. Surgery is required in order to release the synostotic constraint and promote normal calvaria growth. Cranial vault remodeling is the surgical approach to CSS repair at our institution and it involves excision of the frontal, parietal, and occipital bones. The purpose of this article is to describe the post-operative course of infants and children admitted to our PICU after undergoing cranial vault remodeling for primary CSS.

**Findings:**

Complete data was available for analyses in only 82 patients, 44 males (M) and 38 females (F); M: F ratio was 1:1.2. Patients (pts) age in months (mo) ranged from 2 mo to 132 mo, mean 18.2 ±-24.9 mo and weights (wt) ranged from 4.7 kg to 31.4 kg, mean 10.24 ± 5.5 Kg.. Duration of surgery (DOS) ranged from 70 minutes to 573 minutes mean 331.6 ± 89.0 minutes. No significant correlation exist between duration of surgery, suture category, patient's age or use of blood products (P > 0.05). IOP blood loss was higher in older pts (P < 0.05) and it correlates with body temperature in the PICU (P < .0001). Post-op use of FFP correlated with intra-operative PRBC transfusion (P < 0.0001). More PRBC was transfused within 12 hrs-24 hrs in PICU compared to other time periods (P < 0.05). LOS in PICU was < 3 days in 68% and > 3 days in 32%. Pts with fever had prolonged LOS (P < 0. 05); re-intubation rate was 2.4% and MVD were 1.83 days. Repeat operation for poor cosmetic results occurred in 9.7% of pts.

**Conclusions:**

Post-op morbidities from increased use of blood products can be minimized if cranial vault remodeling is done at a younger age in patients with primary CSS. PICU length of stay is determined in part by post-op pyrexia and it can be reduced if extensive evaluations of post-op fever are avoided.

## Introduction

**Figure 1:**
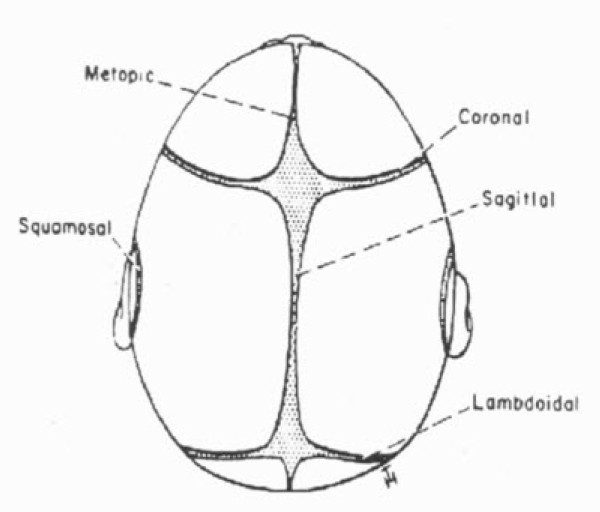
**Cranial sutures in infancy**. Premature closure of any of the calvarial sutures produces a restriction of growth vectors leading to craniosynostosis.

Primary craniosynostosis (CSS) results from the premature closure of calvarial sutures (Figure 1) leading to a deformed calvaria at birth; functional deficits or cosmetic abnormalities can occur later in life if surgery is not performed. Although its etiology is unknown, animal studies point to a local alteration in the expression of growth factors leading to premature fusion of cranial sutures [1,2]. The fused suture restricts growth of the calvaria leading to characteristic deformities typical of primary CSS. The incidence of primary craniosynostosis (CSS) is 1:2000 births and it is usually diagnosed in infancy and early childhood [3]. In comparison, secondary CSS involves all sutures and it is related to growth failure of the entire brain. Although there are many studies in the literature describing the various neurosurgical techniques for correction of deformities associated with craniosynostosis only few studies address the post -operative course and morbidities in the intensive care unit after cranial-vault reconstruction in children with CSS. The purpose of this article is to describe the post-operative management of infants and children admitted to the pediatric intensive care unit (PICU) at our institution after undergoing cranial vault reconstruction.

## Materials and methods

The medical records of patients with CSS who underwent cranial vault reconstructive surgery at Tulane University Hospital between June 1996 and June 2006 were retrospectively reviewed. A waiver of informed consent was granted by Tulane university institutional review board (IRB# 09-16032). The charts were reviewed with regard to demographics, intra-operative blood loss, and duration of surgery. Intra-operative consumption of blood products (crystalloids, PRBC, FFP, 5% albumin and platelets) were also included in the analyses. Post-operative (POP) course in the PICU at the time periods 12 through 36 hours were analyzed with respect to POP blood loss, infusions of blood products and crystalloids (CRYSTS). Also, the length of stay (LOS), mechanical ventilation days (MVD), post-operative morbidities and complications in the PICU were analyzed. Post-operative analysis was limited to first 36 hours in the PICU because this time frame allows for a thorough observation of the impact of intraoperative events on the PICU course; also, majority of our patients were discharged from PICU at or before 36 hours

### Statistics

All data were summarized using descriptive statistics such as mean, median and standard deviation. The analysis of variance (ANOVA) method and/or Kruskal Wallis test on non-parametric method were applied to compare mean values or center of location parameters between different groups. Correlation analysis was used to study relationships of different interest or variables. Where meaningful, the results were presented graphically. The study hypotheses were tested on the 5% level of significance throughout analyses. All analyses were performed using SAS Software (version 9.1 or higher in a Windows format) and Statistical R-Package.

## Results

### Patient demographics

**Figure 2:**
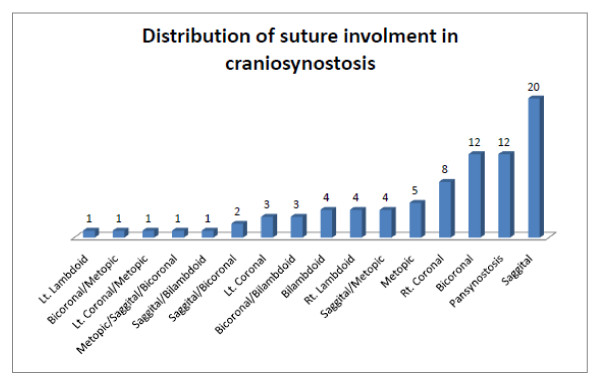
**Total number of each category of suture involved in the different types of craniosynostosis is shown at the top of each bar; coronal and sagittal suture synostoses were predominant in our series**.

82 patients, 44 males (M) and 38 females (F) had complete data for analyses. M: F ratio was 1:1.2. Patients (pts) age in months (mo) ranged from 2 mo to 132 mo, mean 18.2 ± 24.9 mo (Table 1) and weights (wt) ranged from 4.7 kg to 31.4 kg, mean 10.24 ± 5.5 Kg. 77% of patients are from the State of Louisiana while the rest were referred from the contiguous States of Alabama and Mississippi; 76% of patients are Caucasian, the rest are African-Americans or Asians. Primary CSS occurred in 94% of patients (Figure 2) while CSS with associated syndromes occurred in 9.7% of patients (Table 2).

**Table 1:** Age distribution in patients with craniosynostosis at the time of surgery

Age group (months)	Number of patients	%
**3**	4	5

**4-6**	28	34

**7-12**	26	32

**13-24**	10	12

**≥ 24**	14	17

Majority of patients were greater than 4 months at diagnosis; intra-operative blood loss and post-operative morbidities were higher in older patients

**Table 2:** Syndromes associated with craniosynostosis

Syndromes	N = 8
Apert	1

Pierre Robin	2

Goldenhar	1

Turner	1

Dandy-Walker malformation	1

VATER	1

Arnold-Chiari malformation	1

9.7% of our patients had associated syndromes. Incidence of recurrent synostosis is higher in patients with associated syndromes.

### Pre-operative evaluation

Most patients had a developmental screening test (Denver Developmental Screening Test) performed by their general pediatrician before referral to the neurosurgical team. Some patients had lateral skull radiographs and computerized tomography of the head at the discretion of the neurosurgical team. Twenty patients (24.4%) had evidence of developmental delay (DD) based on parental observation and confirmed by clinical assessment of general pediatricians and the neurosurgical team; however, extensive developmental assessment and psychometric testing were not performed.

### Intra-operative course

**Figure 3:**
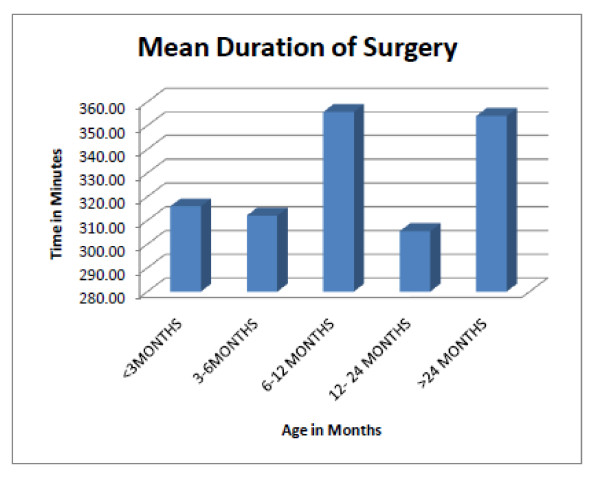
**There was a tend towards longer duration of surgery in older patients but this was not statistically significant (p > 0.005)**.

**Figure 4:**
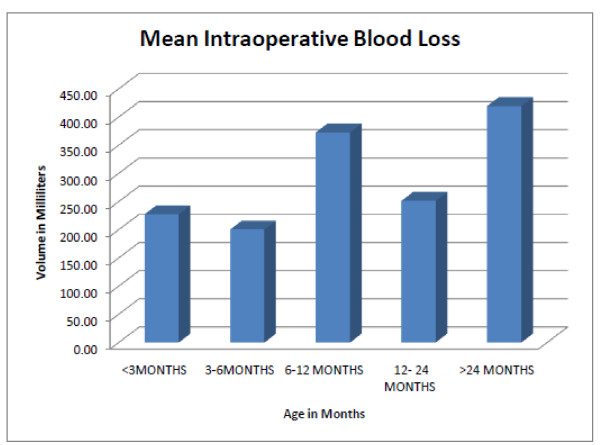
**Blood loss during surgery was higher in older patients (P < 0.05) but a spike in blood loss occurred in the 6 mo-12 mo group because of presence of associated syndromes and a more complicated/protracted surgery in 2 pts in this group**.

The duration of surgery (DOS) ranged from 70-573 minutes (min), mean 331.6 ± 89.0 min. DOS did not correlate (P > 0.05) with age at operation and suture category (Figure 3). Blood loss at surgery (BLS) ranged from 80-2000 mls, mean 320 ± 309.2 mls and it was higher in older pts (P < 0.05); mean BLS in pts < 3 months (mo) is 227 ± 32.0 mls versus 245.55 ± 179.83 mls in pts 4-6 mo. Mean BLS was 396.15 ± 465.81 mls in pts 7-12 mo pts and 412.9 ± 204.27 mls in pts > 24 mo. PRBC transfused at surgery reflects age differences in blood loss at surgery. Increased blood transfusion in the 6 mo-12 mo age group resulted from complications during surgery in two of the patients in this age category (Figures 4). Mean pre-operative hematocrit was 33.4% and mean post operative hematocrit was 33.7% (P > 0.05)

### Postoperative course in the PICU

**Figure 5:**
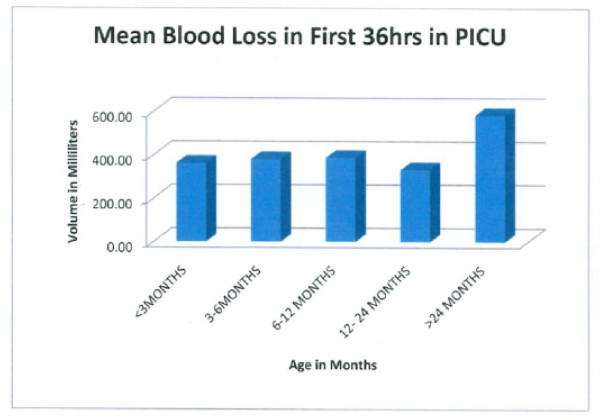
**Post-op blood loss in the PICU was higher in older patients in the time period indicated**.

**Figure 6:**
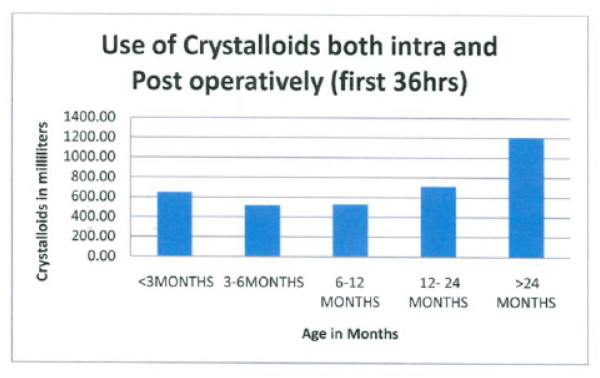
**There was a trend towards increased use of crystalloid intra-operatively and in the first 36 hours in PICU**. This trend reached statistical significance (P < 0.05) and correlated with age.

**Figure 7:**
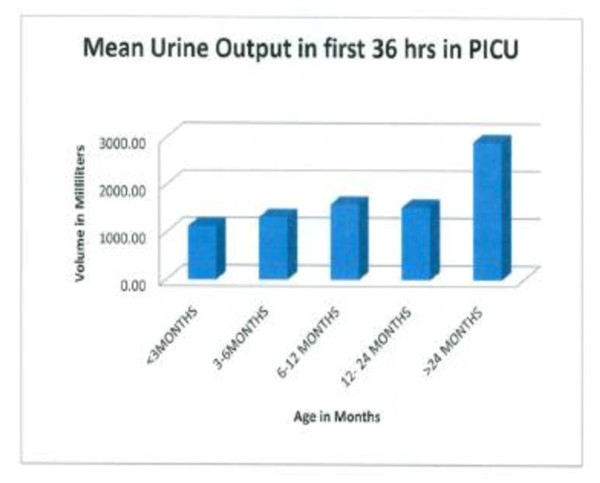
**Urine output was a surrogate marker for intravascular volume status; increased urine output correlates with crystalloid administration (P < 0.001)**.

Volume of blood products transfused in PICU post-op showed significant correlation with volume of PRBC transfused intra-operatively (P < 0.0001). Blood loss and PRBC administered in the PICU through time periods 0-36 hrs (Figure 5) did not show statistical significance when matched with age at surgery or duration of surgery (P > 0.05); however, more PRBC was transfused at 12 hrs-24 hrs of admission to PICU compared to time periods 0-12 hrs and 24 -36 hrs (P < 0.05). Also, post-op use of blood products (FFP, Platelets) correlates with volume of PRBC transfused at surgery (P < 0.0001). Crystalloid infusion in 12 hrs-36 hrs of PICU admission (Figure 6) correlates with age, weight and increased urine output (P < 0.0001). Intravascular volume status is usually restored to baseline by 12-24 hrs of PICU course as shown by increased urine output (Figure 7). Temperature in the immediate PICU course ranged from 36.87°C-37.06°C. Fever (temperature > 38.5°C) occurred in 4 patients but only 1 patient had a positive blood culture and lobar infiltrate on chest radiographs. There was a significant correlation between body temperature on admission to PICU and volume of blood loss in surgery (P < .0001). Presence of fever in the PICU course correlates with prolonged LOS (P < 0. 05). LOS was < 3 days in 68% and > 3 days in 32%; two pts had a LOS in excess of 10 days; one was an ex-premature baby (6 mo old) with congenital heart defects, chromosomal abnormalities and cardiac failure while the other pt was a 6 mo old with sepsis. Both patients had a longer duration of surgery and post-op complications like seizures and respiratory distress requiring re-intubation.

Post-op morbidities like facial edema, hyponatremia, seizures and cardiac rhythm disturbance, especially bradycardia were observed in 6% of pts. Hyponatremia was due to syndrome of inappropriate anti-diuretic hormone (SIADH) in one patient; the cause of hyponatremia was undetermined in a second patient who had associated seizure. Mechanical ventilation days (MVD) were 1.83 days with a 2.4% re-intubation rate. Repeat operation was performed in 9.7% of pts for poor cosmetic results (Table 3). Surgical complications occurred in 2 pts (2.4%); these complications include a thermal injury to the left knee in a 2 mo old and a left forehead encephalocoele in addition to a subdural hematoma in a 4 yr old.

**Table 3:** Number of patients requiring repeat operation for cosmetic and other problems after initial surgery for craniosynostosis

Age at first surgery (suture category)	Age at re-operation	Reasons for re-operation
**5 yrs (sagittal, lambdoid)**	8 yrs	↑ICP, headache, unsteady gait

**11 mo (pansynostosis)**	11 mo**	temporalis muscle re-suspension

**6 mo (rt. Lambdoid)**	4 yrs	bony defect over posterior sagittal sinus

**18 mo (pansynostosis, Crouzon's variant)**	18 mo*	hemifacial reconstruction

**6 mo (bicoronal)**	6 yrs	Facial asymmetry & orbital dystopia

**5 yrs (sagittal/metopic)**	6 yrs	Repair of multiple cranial defects

**1 yr (sagittal)**	7 yrs	cranioplasty for frontal skull defects

**7 mo (coronal synostosis)**	4 yrs	Non-healing coronal incision, craniofacial deformity, repair of Lt encephalocoele

*3 days after first surgery; **4 days after first surgery;

Patients with recurrent synostosis and major surgical complications were 6 months or older at time of initial surgery. Poor cosmetic outcomes appear to be common in patients older than 6 months at the time of initial surgery.

## Discussion

Over 60% of our patients were older than six months at the time of surgery with an average age of 18.2 ± 24.9 months. The older patients in our study consumed more blood products intra-operatively and in the immediate post-op period in the PICU compared to younger ones. In this regard, our result differs from that of Eaton et al [4] who reported increased use of blood products in younger patients in their series. Since the surgical approach was similar in both studies these differences could be due to differences in blood transfusion policies, experience of the surgical team and factors related to anesthesia. The same team of surgeons and anesthesiologist were responsible for all the cases in our series compared to multiple teams in the series by Eaton et al [4]. Overall, the more invasive the surgical approach the more blood products consumed post-operatively. For example, endoscopic assisted repair (EAR) of CSS results in less blood loss at surgery and minimal use of blood products post-op [5,6] because it is a less invasive approach compared to cranial vault reconstruction. The increased use of blood products and crystalloids in the immediate post-operative period (12-24 hrs) in our series was due to the longer duration of surgery (> 6 hrs) and the invasiveness of cranial vault reconstruction compared to EAR. Also, dilutional coagulopathy resulting from massive blood transfusions during surgery could explain the increased use of FFP and platelets (P < 0.05) in the immediate post-op course in our series. Though EAR is a less invasive surgical option for CSS repair compared to cranial vault reconstruction it is not the procedure of choice in older patients and in those with associated syndromes [5,6]. EAR was not the preferred surgical option for CSS repair in our patients because the mean age at presentation in our series was of 18.2 ± 24.9 mo. Another reason why EAR may not be suitable for our patients is the associated cost of EAR because over 80% of the patients in our series have no health insurance and cannot afford the expense associated with mandatory use of helmet for 6-12 months after endoscopic assisted repair. Another major limitation besides cost is poor compliance with use of helmet because it is mandatory to wear it for six months to one year after surgery [5,6].

LOS was longer in our series mean (3.08 days vs. 1 day) compared to what was reported after endoscopic repair [5,6]; however, LOS in our study was comparable to the LOS reported after cranial vault reconstruction for CSS in other studies [7,8]. Facial edema resulting from increased use of crystalloid (Figure 6) and blood products was common 12-24 hrs after surgery and was worse by post-op day two. This observation is similar to the time course for development of facial edema after CSS surgery in other studies [9-11]. Resolution of facial edema usually occurred by 36 hrs of PICU admission in our series because of increased urine output and mobilization of third space fluid (Figure 7).

Fever of unknown origin, which is widely reported as a common post-op morbidity in most series [9-11] occurred in 5% of our patients. However, hypothermia was equally problematic in the first few hours of admission to the PICU, especially in infants. Our results show a significant statistical correlation (P < 0.001) between volume of blood loss at surgery and body temperature changes in the PICU. It is plausible that post-op fever after CSS surgery might be more related to consumption of large volumes of blood products during and after surgery than to an infectious cause because febrile transfusion reactions often complicate massive blood transfusions; on the other hand, hypothermia could result from transfusion of large quantities of refrigerated blood products. Prolonged PICU LOS in some of our patients was related to investigations for fever of unknown origin.

In light of this observation we agree with other investigators [9-11] that an extensive investigation of post-op fever in patients with CSS is ill advised because a treatable cause is hardly identified. We no longer perform extensive sepsis work-up in our patients. It is possible that this policy might reduce LOS in PICU because post-up fever was one of many factors that determine prolonged PICU LOS in this series

Postoperative seizures due to fever and hyponatremia occurred in two of our patients (2.4%). This is comparable to the 2.9% incidence of seizures reported in published reports [12]. Hyponatremia resulting from cerebral salt wasting (CSW) or SIADH has been reported in CSS patients after surgery [12,13]. SIADH was diagnosed in one of two patients with hyponatremia in our series but the cause of hyponatremia was not identified in the second patient. Fluid restrictions resulted in resolution of SIADH in our patient and avoidance of hypotonic fluids reduced the incidence of hyponatremia in the post-operative period.

The risk of infection is higher following an invasive surgical technique like cranial vault reconstruction compared to a minimally invasive approach like endoscopic assisted osteotomies [14]. Despite this risk the rate of infection was only 2.4% in our patients. The low infection rate in our series could be related to use of resorbable plates by our surgical team, a practice that have been shown to reduce infectious complications when compared to use of metal screws. Our patients did not develop the typical complications like dural tears, CSF leaks and subcute hematomas seen after cranial vault reconstruction [15,16]; however, thermal injury occurred in a 2 month old and a 4 yr old had a non-healing coronal incision complicated by a subdural hematoma and frontal encephalocoele. About 90% of our patients had good cosmetic results and only eight of eighty-two patients (9.7%) had repeat operations for poor cosmetic outcomes (Table 3). Though the mean age at surgery in our study was 18.2 months only one of our patients required re-operation for recurrence of craniosynostosis. This is remarkable because older age at the time of surgery is a known risk factor for re-synostosis and poor cosmetic outcome [17]

A major limitation of this study is that it is a retrospective analysis of data spanning a period of ten years therefore our results might not reflect more recent surgical outcomes in patients with craniosynostosis. For example, a less invasive innovative surgical technique like endoscopic assisted repair of CSS results in less blood loss and reduction in LOS. However, there are indications for cranial vault reconstruction surgery in some subsets of patients with craniosynostosis therefore a clinical description of common post-op issues associated with this technique remains invaluable to pediatric clinicians.

## Conclusions

We report our experience with the postoperative care of patients with craniosynostosis at our institution over a period of ten years. Our results indicate that a good outcome can be accomplished in the post-op period by a multidisciplinary team of providers despite the complexity and risks associated with cranial vault reconstructive surgery in patients with CSS. Despite the development of an alternative surgical technique like endoscopic assisted repair for CSS patients who present early for surgery, cranial vault reconstruction remains a surgical option for older patients with CSS including those with syndromic synostosis. Therefore, a description of the intensive care unit course and post-operative management issues unique to some subsets of CSS patients who require cranial vault reconstructive surgery might add to the existing pool of valuable clinical data available to pediatric clinicians.

## Abbreviations

ANOVA; BLS; CRYSTS; CSS; CSW; DOS; FFP; IRB; LOS; MVD; PICU; POP; PRBC; SIADH.

## Competing interests

The authors declare that they have no competing interests.

## Authors' contributions

OA, DS and SS worked on the data abstraction, data analysis and overall structure of the manuscript. JW, DJ and EF helped in revising and critique of the manuscripts. All authors read and approved the final manuscript.
